# Interdisciplinary Trauma-focused Therapy and Return-to-work Support for A Police Officer with Work-related PTSD: A Case Study

**DOI:** 10.1007/s10879-022-09547-6

**Published:** 2022-07-04

**Authors:** Iris Torchalla, John Killoran

**Affiliations:** West Coast Resiliency Centre, 214-3195, V6H3K2 Granville St, Vancouver, BC Canada

**Keywords:** Posttraumatic stress disorder, Police officers, Trauma-focused psychotherapy, Occupational therapy, Return to work

## Abstract

Police officers carry a high risk of exposure to traumatic events in their everyday work duties and are at an increased risk for work-related posttraumatic stress disorder (PTSD). Practitioners lack clear guidance on how to support these individuals to facilitate both mental health recovery and return to work, particularly for those receiving treatment in the context of a claim with a workers’ compensation board. The following case study describes the treatment of a female police officer who had experienced numerous traumatic events over the course of her career, and subsequently filed a claim with the workers’ compensation board of British Columbia. She was referred to an interdisciplinary program that involved both psychology and occupational therapy interventions, including a trauma-focused cognitive behaviour therapy intervention followed by a gradual return to work. The outcome suggests that intensive, interdisciplinary trauma-focused treatment is a promising approach for supporting police officers with their recovery and return to work.

## Introduction

Workplace trauma as a risk for both primary and secondary traumatization has increasingly been acknowledged by workers’ compensation boards, scientists, and in the diagnostic criteria for posttraumatic stress disorder (PTSD). Police officers carry a particularly high risk of exposure to traumatic events, and a meta-analysis estimated that 14.2% of police personnel worldwide have PTSD (Syed et al., 2020). A number of empirically supported interventions are now available to treat individuals with PTSD. Numerous clinical practice guidelines make strong recommendations for trauma-focused cognitive behaviour therapies (Hamblen et al., 2019). This term refers to treatment protocols that involve processing the trauma memory to resolve trauma-related feelings, make meaning of the experience, and integrate corrective information. Some protocols also include in vivo exposure which involves confronting feared and avoided situations without escaping or using safety behaviours (Blakey & Abramowitz, 2019). Meta analyses have demonstrated that trauma-focused cognitive behaviour therapies achieve medium to large effect sizes for PTSD symptom reduction and loss of diagnosis (e.g., Morina et al., 2021). A randomized controlled study among first responders found that trauma-focused cognitive behaviour therapy resulted in large reductions of PTSD severity, and 56–59% of participants no longer met criteria for PTSD at the 2-year follow up (Bryant et al., 2021).

If a claim of work-related PTSD was accepted by a workers’ compensation board, the individual is entitled to receiving treatment for their PTSD. Typically, expectations are related to both symptom reduction and resumption of work. However, research reporting return-to-work outcomes of interventions for individuals with work-related PTSD has remained scant (Torchalla & Strehlau, 2018). In addition, despite the strong evidence for the effectiveness of exposure-based psychotherapies, they remain underutilized by community-based clinicians (Pittig et al., 2019). Barriers include clinicians’ beliefs that exposure might exacerbate symptoms and lead to treatment non-compliance and dropout or a disruption of the therapeutic relationship (Michael et al., 2021), or that it does not work for complex cases and individuals with cumulative trauma or trauma-related feelings other than fear (Cook et al., 2018).

Information regarding the treatment of PTSD in police officers is important because they often present with a number of these perceived challenges. For example, cumulative traumatic experiences are common in this profession (Carleton et al., 2019). Furthermore, police officers are trained to remain calm in stressful situations and to supress emotional responses at the time of an event. This emotional detachment is valuable for their functioning at work, but may result in them being unwilling or unable to access and describe their emotions even when they are off duty (Lennie et al., 2020). Many first responders also struggle with the impact of morally challenging experiences that might trigger emotions beyond fear-based distress. For example, they frequently report a sense of failure to protect members of the public, and their moral outlook might be shattered by the repeated witnessing of cruel acts by others (Bryant, 2021).

In summary, although effective interventions for PTSD exist, many aspects of work-related PTSD have not been adequately researched or translated into practice, and practitioners lack clear guidance on how to support these individuals to facilitate both recovery and return to work, particularly workers with highly stressful jobs and complex presentations such as police officers, and those receiving treatment in the context of a workers’ compensation board claim. It has been suggested that clients who struggle to return to work after workplace trauma might benefit from interdisciplinary treatment that includes counselling for trauma processing as well as occupational therapy to support graded work-related exposures (Torchalla et al., 2019). The following report describes the treatment of a police officer who participated in a psychotherapy program and subsequent gradual return to work as part of her claim with WorkSafeBC, the workers’ compensation board of British Columbia. The report aims to demonstrate how interdisciplinary trauma-focused therapy can facilitate PTSD recovery and return to work after police work-related trauma, and to highlight specific components that may be helpful for treating police officers or other first responders with work-related PTSD.

## Referral Process

WorkSafeBC accepts claims for mental disorders that arise from traumatic work events. First, the worker will be referred to a diagnostic assessment, to determine if they have a mental disorder which was predominantly caused by work-related factors. If a claim is accepted, benefits such as wage-loss compensation and health care treatment will be provided, including referral to specialized trauma recovery programs. WorkSafeBC has contracted multiple clinics across the province to deliver these services, including the current clinic. In this program, each client works with a team consisting of a psychologist and an occupational therapist, who collaborate closely in delivering trauma-focused, exposure-based psychotherapy and return-to-work support. If a worker does not reside within driving distance to the clinic, WorkSafeBC will cover the costs of travel and accommodation to facilitate participation in the program. During the COVID-19 pandemic, clients participated in telehealth sessions if this mode was considered clinically appropriate. Potential clients first participate in an intake assessment with one of the team psychologists to determine their eligibility for the program. The treatment program is 8 to 12 weeks long (the exact duration depends on the client’s progress). If the client progresses well and feels ready to attempt a return to work to their pre-injury position, they participate in a 4-8-week gradual return to work plan which is monitored by the team. If the client is not able to return to their pre-injury position, they will be discharged with recommendations for restrictions and/or limitations to move forward with vocational rehabilitation or further treatment.

## Case Introduction

### Client Background and History

Susan (pseudonym), a Canadian female in her fifties, was referred to the clinic after being diagnosed with PTSD as a result of her work duties. She reported a happy childhood without abuse or neglect. She is married and has two adult children. She had been working as a police officer in various units for over 20 years. Her claim was accepted based on specific recent incidents, but she reported having experienced and witnessed many traumatic events over the course of her career, such as exposure to victims of homicide, suicide, abuse, assaults, and accidents; her life being threatened; and dealing with aggressive and violent individuals. She was involved in a shooting early in her career. She subsequently struggled emotionally though did not make the connection to the work event. Eventually she sought help from a psychologist who diagnosed her with PTSD. Her mental health improved following psychoeducation and discussing anxiety management techniques. Subsequently, to cope with work-related stressors, she would take her holidays or take a day off if needed, so that she could “get back on her feet” and continue working. She also attended critical incident debriefs at work and sought additional assistance from her physician and her psychologist. Psychotherapy involved supportive counselling and discussion of personal stressors (e.g., parenting concerns, interpersonal relationships). She signed off work during a wire tap operation, where she overheard domestic violence occurring and listened to individuals making suicide plans. She filed a claim with WorkSafeBC and started seeing a psychologist. She found this helpful but noted “I needed deeper help.” Hence, she was referred to the trauma recovery program. Treatment was delivered by a Doctoral level psychologist and a Master’s level occupational therapist.

### Intake Interview

At the time of her intake interview, Susan had been off work for seven months. She had requested an in-person assessment because she felt this would facilitate building trust. She appeared tense for most of the session. She provided extensive information about stressful work events and had at times difficulty containing her memories. She reported experiencing intrusive auditory memories associated with the wire tap operation, visual memories related to her work with a victim of domestic violence, and brief visual “flashes” of other seemingly random calls. She often woke up at night with her heart pounding and sweaty palms, and lay awake thinking about work. She was feeling sad, anxious and guilty, and often found herself ruminating about events for which she blamed herself (e.g., not having done enough to prevent deaths). She tried to push her memories and feelings away because they seemed overwhelming and she did not know what to do with them. She avoided listening to the news, getting into arguments with others, watching certain TV series and going to places that reminded her of calls. She endorsed increased irritability, being impatient with her family, and being tense around people. She reported feeling constantly on guard and on the edge, feeling easily startled, crying more than usual, and having difficulty concentrating. She had developed hives and headaches in response to stress, was grinding her teeth and was wearing a mouthguard at night because of this. She had stopped participating in sports activities, and she used to be an outgoing person but now she preferred to be alone. She denied having ever had any suicidal ideation. She reported occasional alcohol use but no tobacco or recreational drugs use. At the time of the intake, Susan stated that she would like to retire from policing upon treatment completion.

### Psychometric Assessment

PTSD and depressive symptoms were measured at intake, every four weeks during treatment, at the end of the gradual return to work, and at the 9-month follow-up. PTSD symptoms were assessed using the PTSD Checklist for DSM-5 (PCL-5; Weathers et al., 2013), which measures symptom severity on 20 items using a 5-point scale, resulting in a score between 0 and 80. A cut-score of 31–33 points is indicative of a probable PTSD diagnosis. Depressive symptoms were assessed using the Patient Health Questionnaire (PHQ-9; Kroenke & Spitzer 2002), which measures the severity of depression on 9 items using a 4-point scale. The total score can range from 0 to 27, reflecting none-minimal (0–4), mild (5–9), moderate (10–14), moderately-severe (15–19), and severe (20–27) depression. A cut-score of > 9 has been recommended for detecting clinically significant depression. The Inventory of Psychosocial Functioning (IPF; Bovin et al., 2018) was administered at intake, the end of the gradual return to work, and the 9-month follow-up. The IPF is an 80-item measure to assess difficulties in functioning in seven life domains using a 7-point scale. Clients skip domains that do not apply. The responses yield an index of functioning between 0 and 100 for each domain, reflecting no (0–10), mild (11–30), moderate (31–50), severe (51–80), and extreme (81–100) impairment.

## Treatment Planning

A shared decision-making approach is recommended for PTSD treatment where clients and clinicians review treatment options together to determine which intervention best meets the client’s needs and preferences (Hamblen et al., 2019). Susan received information about prolonged exposure (Foa et al., 2019), cognitive processing therapy (Resick et al., 2016), and cognitive therapy for PTSD (CT-PTSD; Ehlers et al., 2005). She chose CT-PTSD. The underlying model suggests that PTSD develops when a person interprets a traumatic event in a way that produces an ongoing external (e.g., “The world is dangerous”) and/or internal (e.g., “I’ve let myself down”) sense of threat. The sense of threat is maintained by three processes: First, the person has developed excessive negative appraisals of themselves and others. Second, because the trauma memory is disjointed and poorly elaborated due to avoidance, the person is unable to access information that could correct their negative appraisals and make sense of their experiences. Third, the individual uses cognitive and behavioural strategies that reduce their sense of threat (e.g., memory suppression, rumination, safety behaviours, avoidance) but prevent reappraisal of the events. CT-PTSD aims to elaborate and update the trauma memory, modify negative appraisals and make meaning of the event, and reduce unhelpful strategies. Core interventions are psychoeducation, an individualized case formulation, reclaiming your life assignments, trauma processing, trigger discrimination and exposure to triggers.

Susan’s treatment consisted of 12 weeks of CT-PTSD involving an initial joint session with the team, followed by two occupational therapy sessions and two psychology sessions per week. Most sessions were completed via secure videoconferencing platform due to the recent Covid-19 outbreak, but the first two trauma processing sessions with the psychologist and several exposure sessions with the occupational therapist were held in person. Working together as a team, the psychologist provided education and the case formulation and then focused primarily on trauma processing. The occupational therapist reinforced education, and then focused on improving functioning in the areas of self-care, leisure and productivity, supporting Susan in confronting situations and activities that were triggering or being avoided as a result of her work experiences, and developing and overseeing the gradual return to work plan.

## Treatment Structure

### Psychoeducation and Individualized Case Formulation

Both the psychologist and the occupational therapist provided education to help Susan understand PTSD, normalize her symptoms, and instill hope for recovery. Aspects of PTSD that are particularly relevant for first responders were also addressed, such as the need to suppress emotional responses while on duty to perform in a safety critical role; the police culture; dynamics of accumulative stressful events; trained safety behaviours (e.g., increased vigilance, scanning for threats and planning escape); and the range of emotional reactions associated with moral distress. The psychologist also worked with Susan to develop an individualised case formulation. This included a depiction of her experiences of current threat (e.g., feeling like a failure), and identifying the processes that maintained it (e.g., her high moral standards, emotional suppression at the time of the events, specific cues that triggered her symptoms, her tendency to ruminate and to avoid exploring her memories and emotions, resulting in disjointed memories and unresolved emotions which maintained her negative beliefs about herself). Following this discussion, the psychologist and Susan developed strategies to drop rumination and allow memories and emotions to come and go instead of pushing them away.

### Reclaiming Your Life Assignments

Like many individuals with PTSD, Susan felt that she had permanently changed for the worse and had become a different person as a result of her work experiences. She used to be social, curious and an avid athlete, but had given up many activities and relationships that had been a significant part of her life. The occupational therapist discussed with her what she could do to reclaim previously valued and enjoyed activities. Susan’s goals included increased socialization with friends and enjoyable activities with her husband, increased physical activity, regular quiet activities such as reading, improving her proficiency in a second language, improved sleep quality, and regular mindfulness practice. Susan was initially ambivalent about returning to work, though the team encouraged her to focus on treatment and make this decision at the end of program. Behavioural goals were discussed weekly and broken down into more specific and targeted goals. Susan and the occupational therapist agreed on achievable first steps in these areas using a weekly planner and Susan completed assigned activities between sessions. Sleep hygiene was also discussed and Susan implemented several strategies, including establishing a sleep routine (a gratitude journal, worry journal, sleep and meditation apps), setting a consistent sleep and wake schedule, regular sunlight exposure in the morning, and avoiding naps during the day.

### Trauma Processing

Trauma processing following CT-PTSD protocol involves accessing the trauma memory, identifying meanings that maintain a sense of threat, and integrating updating information into the trauma account. Trauma-related discussion began by drawing a timeline of events that covered the entire period of Susan’s career. Three events were identified that were highly distressing as well as representative of diverse emotional experiences (fear, sadness, guilt, moral distress): working with a victim of domestic violence; dealing with an aggressive man who held his spouse and child captive; and the wire tap operation. Accessing the trauma memory in CT-PTSD can be completed using imaginal reliving or narrative writing. Susan chose narrative writing which involves writing a detailed account of the trauma while engaging with the associated emotions.

Trauma processing began with addressing her work with an indigenous woman who was the victim of excessive domestic violence. Susan wrote an account of the event as an assignment and read it out loud to the psychologist during sessions. Although she had a strong grasp of the rationale for experiencing and expressing emotions during therapy and did not share commonly held beliefs that discussing emotions is unacceptable, she had difficulty recognizing when she was distraught, struggled with describing and naming her feelings, and tended to focus on factual information that lacked description of her inner experiences. She appeared detached from her emotions while reading the account. Therefore, time was spent slowing down the reading, alternating reading with pausing to scan her body for physical sensations that might be suggestive of emotional experiences, and exploring the associated thought and feelings. Early on she still tended to focus on other people’s experiences (e.g., the victim’s or the perpetrator’s feelings), but her awareness of her emotional and physical reactions increased over time.

The next step involved identifying the moments associated with the highest distress and negative appraisal (the ‘hot spots’). Susan’s hot spots involved disturbing images of the assaulted victim, accompanied by feelings of horror and helplessness and negative appraisals (“the world is cruel”, “I am powerless and my work is meaningless because I could not prevent this”). Another hot spot involved the victim crying and in distress, blaming Susan for pushing her to go to court, accompanied by feelings of guilt and negative self-appraisal (“I am cruel and a terrible person”). The trial went well, but months later the victim died from an illness, resulting in feelings of guilt, sadness and numbness, associated with a sense of defeat, and thoughts that she has failed to protect the woman and has let the woman and her family down. After identifying the hot spots and allowing herself to feel the associated emotions, updating information was collected via guided discovery and Socratic questioning. This involved considering the situation from different perspectives, reviewing the context and circumstances and the steps she did take to help, discussing what she knows now but did not know at the time, and exploring her expectations and intentions related to her actions. This enabled her to put the meaning of the hot spot into perspective (e.g., “There are cruel people in the world but I can still do my part to make the world a better place”), consider knowledge that was unavailable at the time of the event (“I am glad I pushed her to go to court even though it was hard for her, because afterwards she was feeling so much stronger”) and reflect on evidence for and against her negative appraisal (“I supported her as much as I could but there was nothing I could have done to prevent her death”). A collaborative working style is essential for these discussions, and the psychologist has to assume a perspective of curiosity rather than trying to prove the client’s perspective to be wrong. After every session, Susan incorporated the updating information into her narrative, which she read to the psychologist in the following session. To find closure, Susan and the occupational therapist went to one of the sites associated with the woman. In addition, she wrote a letter to the woman, which she read to the psychologist and subsequently burned in a ritual with her husband.

Susan and the psychologist then proceeded to the second work event: a call involving a violent man who was high on drugs and attacking his spouse and their newborn. Susan first provided a detailed narrative of the event. Because she had suppressed her feelings at the time of the call in order to be able to do her job, time was spent to explore her current emotional and physical reactions while she was remembering the event. The dominant emotion was fear for the victims, herself and her partner, and hot spots were associated with three moments: the perpetrator dragging his spouse by the hair, whose face was covered in blood; the perpetrator holding the baby; and Susan and her partner fighting with the perpetrator to take him down. The personal meaning of these moments was explored (e.g., “What was the worst thing about this?”, “What did you think was going to happen?”, “What did this mean to you at the time?”, “What does this mean to you now?”), along with exploring current physical and emotional reactions. In the next step, updating information was identified (e.g., the woman survived; Susan was able to secure the baby unharmed; another police officer arrived on scene and helped them to take the perpetrator down), which was subsequently incorporated into the trauma account. To find closure, Susan visited the site of the call with the occupational therapist.

The third event involved participating in a high-pressure wire tap operation where Susan overheard domestic violence occurring and listened to delinquents making suicide plans. This was associated with feelings of fear, horror, helplessness, and anger, and various negative appraisals related to moral distress (e.g., “I am a horrible person to let this happen without doing anything about it”) as well as a general sense of inadequacy (“I can’t do this, I am a failure”). Identifying hot spots, exploring personal meanings and identifying and incorporating updating information was completed using the same strategies as described for the other two work events.

### Exposure to Triggers

Exposure to triggers following CT-PTSD protocol involves trigger identification, trigger discrimination, confronting triggers, and visiting trauma sites while dropping safety behaviours. Discussions were held to create a list of triggering and avoided situations. A subjective units of distress (SUDs) scale was used to rate the situations on a 0–10 scale based on Susan’s anticipated distress in each situation. The hierarchy included situations that she found to be generally stressful (e.g., crowded locations such as transit during rush hour and large malls) as well as several places she had avoided due to experiencing difficult calls. Safety behaviours were also identified and coping strategies reviewed. All of this was primarily completed by the occupational therapist but the psychologist added information emerging from their sessions.

Susan completed ten exposures accompanied by the occupational therapist, and additional exposures independently. To prepare her for returning to work it was important to identify and confront all relevant situations, to increase her confidence in her ability to function in these places. During exposures, the occupational therapist facilitated trigger discrimination by encouraging Susan to describe what was different between ‘then’ (the traumatic event) and ‘now’ (the reminder). He also assessed Susan’s distress level regularly using the SUDs scale, coached her through breathing and grounding strategies, and highlighted safety behaviours. However, when working with police officers, therapists need to be aware that many of their avoidance and safety behaviours are adaptive and based on training and experience to keep them safe. In the following sessions, Susan discussed what she had learned from the exposures and reflected on themes such as civilian versus police officer level of vigilance; noticing civilians as regular people rather than threats to her safety; and accepting that there is some level of risk involved in police officers’ daily activities, but that it is important to not overestimate the risk.

Susan and the occupational therapist also completed site visits to places that were associated with the events that Susan was processing with the psychologist. For example, they went to the house where Susan had taken down the violent man, and she was able to highlight differences in the neighbourhood (‘then’ versus ‘now’), recognize the house where the event had occurred and walk around the property to get a different perspective. She experienced physical and emotional reactions during exposures though had good insights during each outing and made excellent improvement over time. Collaboration between the psychologist and occupational therapist involved discussion around coordinating the timing of exposures with trauma processing to facilitate closure. However, it should be noted that trauma processing does not always need to occur prior to in vivo exposure.

### Gradual Return to Work

Although Susan had initially informed the team that she wished to retire from policing following therapy completion, as her treatment progressed, she reported increased interest in returning to work as a police officer. Graded exposure to work-related triggers was initiated mid treatment and was an important precursor to returning to work. A seven-week gradual return to work plan was then created. Susan was an additional (supernumerary) worker on shift, so that scheduling shifts was flexible and work duties and hours could be adjusted and gradually increased, and to allow her to take micro-breaks for practicing symptom management strategies and pacing her duties as needed. This was particularly important as police officers hold safety sensitive positions and the team needed to assess how she responded to various work-related stressors. A relapse prevention plan was also created which included strategies for maintaining a healthy routine and work-life balance as she transitioned back to work.

The occupational therapist met with Susan weekly throughout the gradual return to work, and the psychologist met with her twice for check-ins and for the firearm clearance. Susan continued to report some anxiety early on and worried about her ability to manage stressors, whether she would ultimately be successful in returning to police work, and how to best navigate conversations with colleagues regarding her absence. The occupational therapist normalized and validated her anxiety, spent some time role-playing and developing strategies for navigating conversations, and reviewed stress management strategies and her relapse prevention plan. Subsequently Susan presented in good spirits, expressed no concerns about her gradual return to work, and reported feeling positive about being back in the workplace.

## Outcomes

Throughout her time in the program, Susan never missed a session, demonstrated excellent engagement and participation in all sessions, diligently completed her homework assignments, and made steady progress with all aspects of her therapy. She demonstrated an increased awareness of her inner experiences and ability to express her emotions. She reported improvements in her mood, sleep, anxiety and ability to relax, and she no longer experienced intrusive memories, ruminated about the events and her perceived failures, or avoided reminders. Her hives, headaches, and teeth grinding subsided. At the end of the gradual return to work, she reported being able to perform her work well with full duties and full hours which was confirmed by her employer. She was discharged from the program as fit to return to work without restrictions or limitations. She noted a plan to retire, though not in the immediate future.

### Psychometric Assessment

Susan’s psychometric scores at each assessment time point are presented in Table 1. At intake, her responses indicated clinically significant PTSD and moderately-severe depressive symptomatology, and significant impairment in her daily functioning. Her scores steadily improved over time. From week 8 onwards, her PCL-5 score was below the cut-score for a probable PTSD diagnosis and her PHQ-9 score was below the cut-score for clinically significant depression. At discharge, her scores indicated minimal PTSD and depressive symptomatology, excellent functioning in all life domains measured, and no impairment in her ability to perform her work. She was able to maintain her improvements through the 9-month reassessment.

**Table 1:** Susan’s scores on symptom and functional outcome measures

Assessment time point Measures	Intake	Week 4	Week 8	Week 12	Discharge	9-month follow up
PCL-5	50	42	25	8	6	3
PHQ-9	16	10	8	2	1	0
IPF subscales: Romantic relationship Family Friendships/Socializing Work Self-care	25.76 38.10 60.42 N/A 50.00				7.58 4.76 2.08 0.79 8.33	4.55 4.76 2.08 N/A 0.00
PCL-5: PTSD Checklist for DSM-5 (score range: 0–80); PHQ-9: Patient Health Questionnaire-9 (score range: 0–27); IPF = Inventory of Psychosocial Functioning (score range: 0-100).						

### Follow-Up and Client Perspective

The psychologist met with Susan via telephone nine months after her discharge. Susan shared that she continued to enjoy working following completion of her gradual return to work. She had retired 1.5 months prior to the follow-up appointment. She noted she has been able to maintain her mental health, and was able to participate in her retirement party at the department in good spirits and receive her service award. As part of the informed consent process for this article, the team had offered Susan the opportunity to describe her own perspective of the treatment. In her account, she commented on the challenges related to exposure-based treatment components and highlighted the importance of the therapeutic relationship:

I knew my greatest need was to dig deep into memories that had ripped into my soul over time. I also needed to develop full trust with my therapists to share such darkness and personal struggles. My first session with my psychologist, Dr. Torchalla was especially important to me. My heart was pounding and I was tense and not myself. My hopes for recovery were riding on this relationship. I was treated with such respect, dignity, and kindness that I had hope for finding my way again. Dr. Torchalla gently and persistently encouraged me to write and speak about experiences that were impacting me. The sessions were intense and challenging on one hand but enlightening on the other hand. At the end of one series of sessions, I wrote a letter to the person who had died unexpectedly after an investigation. To help me let go of this painful memory, I burned the letter with sage. Dr. Torchalla had cut sage from her own garden and gave it to me for this purpose! The symbolism of this, along with that of the action taken, has impacted me immensely. It has been the only way I was able to put this matter to rest in my mind and heart over a twelve-year time period. After deep discussion over another difficult incident, I used another strategy to put a matter to rest that had a long-term impact on me. That also worked well. At the same time, I was also engaged in sessions with John, the occupational therapist. His approach to treatment was very pro-active, which I liked, and worked hand in hand with my psychology sessions. We went to places that I had spent time in while working and that held dark memories. I hadn’t realized I avoided them as much as I did. John helped me recognize the impact I felt while there, and view them from today’s perspective. The negative feelings are no longer an issue when I attend those places and I now have an internal dialogue that helps me keep it that way. This brings me hope so that I look forward with a positive mindset to my life ahead.

## Discussion and Conclusions

Intensive PTSD treatments with active-duty military, and more recently, first responders have gained increasing empirical support for reducing trauma-related symptoms. The current case report extends this body of knowledge by using an interdisciplinary intervention that includes evidence-based psychotherapy to facilitate both PTSD recovery and return to work. In this program, existing PTSD therapy protocols are adapted to the workers’ specific needs and clinical presentation in various ways: Including an occupational therapist in the treatment of work-related PTSD is beneficial for addressing the increased functional impairments of individuals who are off work (e.g., due to lack of structure, routine and social interactions and decreased necessity to leave the house). Close collaboration between the occupational therapist and the psychologist allows for both reiteration and more in-depth exploration of therapy components, and enables the team to address multiple facets of treatment concurrently. Because the occupational therapist took over the ‘reclaiming your life’ assignments and exposure to triggers, the psychologist was able to focus primarily on trauma processing. As such, Susan could process several events involving a range of emotional experiences, both typical for police officers and other first responders. Early engagement in enjoyable and relaxing activities also assisted her with improving her mood and ability to tolerate the distress that is associated with the trauma-focused treatment components. Furthermore, although therapy protocols often include recommendations that clients engage in exposure to triggers and avoided situations between sessions, they provide little guidance on how to support individuals who struggle with exposure assignments. Because of her long career there were many places in the city that Susan avoided, and confronting some of them together with the occupational therapist diminished her sense of overwhelm. Close communication between the psychologist and occupational therapist was beneficial for coordinating the site visits with trauma processing to facilitate closure.

Interdisciplinary treatment programs are increasingly recommended for individuals who did not recover after receiving standard PTSD treatment. For example, Martinmäki and colleagues described a day clinic program for police officers, which took place on one day per week for 9 months and included trauma processing, sociotherapy and psychomotor therapy. At the end of the program, 47% of participants had improved or recovered from their PTSD symptoms (Martinmäki et al., 2021). However, the program did not include a return-to-work component. Existing treatment manuals usually lack information on how to address return to work, and the few studies that did modify therapy protocols to facilitate this were conducted with workers who had experienced a single trauma, most notably workers with industrial injuries (see Torchalla & Strehlau 2018 for an overview). Occupational therapists are trained in functional capacity evaluation and thus are well qualified to address work-related psychological impairments and return to work. Consultation with the psychologist prior to and during the gradual return to work process can assist with determining treatment strategies and appropriate work duties. The psychologist can also help monitoring the client’s function during the plan (even though this was not necessary with Susan). Additionally, the team can collaborate to support the worker through creating a relapse prevention plan, reviewing coping strategies, challenging unhelpful beliefs, normalizing symptoms, processing unforeseen circumstances, and managing setbacks.

The integrative treatment was comprehensive and, for Susan, very effective. Like many police officers, she was highly motivated to recover and she engaged in her therapy and the associated assignments with much of the same commitment and diligence as she approached her work duties. This was likely a significant factor in her recovery as adherence to homework is associated with better PTSD treatment outcomes (Barawi et al., 2020). Susan also identified the therapeutic relationship as important for her recovery, which is in line with research suggesting that the therapeutic alliance is an essential element of any effective intervention. For example, in a study using CT-PTSD, a strong working alliance at the very start of treatment predicted greater symptom improvement at the end of treatment (Beierl et al., 2021). A collaborative approach between the client and the team is imperative, where therapists are compassionate, patient, and non-judgmental and create an atmosphere in which clients can safely express feelings of fear, anger, horror, guilt, shame, frustration or sadness. Handling clients’ distress calmly and with non-judgmental curiosity is helpful for building trust and rapport, and after such experiences many police officers report that sharing their trauma memory has been relieving and validating.

Limitations of this case study include its generalizability and the limited follow-up time period. Despite this, it suggests that interdisciplinary, trauma-focused treatment with police officers who are off work with PTSD resulting from cumulative traumatic experiences is effective to support them with their recovery and return to work to an inherently stressful job. Although resource intensive and expensive, it might help workers return to work earlier than less intensive treatment. Larger studies are needed to better understand how to support police officers with PTSD recovery and a sustainable return to work. Studies are also required to infer recommendations how to best support police officers who are not able to return to work to their pre-injury position. Furthermore, prevention is essential. Even though Susan had significant training to manage stressful situations on the job, she had received little training on mental health. Integrating education about first responder stress, coping strategies, self care and the importance of talking about difficult experiences into police departments in a more ongoing, even preventive way could be beneficial. Working with police officers who present with PTSD can be very rewarding for clinicians, and it is important that they acquire the appropriate skills because this will benefit not just the officers but the communities they serve as well.
